# A multicentre benchmark dataset for comprehensive landmark-based fetal ultrasound biometry

**DOI:** 10.1038/s41598-026-47854-3

**Published:** 2026-04-14

**Authors:** Chiara Di Vece, Zhehua Mao, Netanell Avisdris, Brian Dromey, Raffaele Napolitano, Dafna Ben Bashat, Francisco Vasconcelos, Danail Stoyanov, Leo Joskowicz, Sophia Bano

**Affiliations:** 1 Department of Computer Science and UCL Hawkes Institute, University College London, London, WC1E 6BT UK; 2 Tel Aviv Sourasky Medical Center, Sagol Brain Institute, Tel Aviv, Israel; 3 School of Computer Science and Engineering, The Hebrew University of Jerusalem, Jerusalem, Israel; 4 Fetal Medicine Unit, Women’s Health Division, UCLH NHS Foundation Trust and Elizabeth Garrett Anderson, Institute for Women’s Health, University College London, London, UK; 5 Sagol School of Neuroscience and Gray Faculty of Medical and Health Sciences, Tel Aviv University, Tel Aviv, Israel

**Keywords:** Fetal ultrasound, Biometry, Landmark detection, Deep learning, Domain shift, Multicentre dataset, Benchmark, Anatomy, Computational biology and bioinformatics, Engineering, Health care, Mathematics and computing, Medical research

## Abstract

Accurate fetal growth assessment from ultrasound (US) relies on precise biometry measured by manually identifying anatomical landmarks in standard planes. Manual annotation of landmarks is time-consuming, operator-dependent, and sensitive to variability across scanners and sites, limiting the reproducibility of automated approaches. There is a need for multi-source, annotated datasets to develop artificial intelligence-assisted fetal growth assessment methods. To address this bottleneck, we present an open-access, multicentre benchmark dataset of fetal US images with expert anatomical landmark annotations for clinically used fetal biometric measurements. These measurements include head biparietal and occipitofrontal diameters, abdominal transverse and anteroposterior diameters, and femoral length. The dataset contains 4,513 de-identified US images from 1,904 subjects acquired at four clinical sites using seven different US devices. We provide subject-disjoint train/test splits, evaluation code, and baseline results to enable fair and reproducible comparisons of methods. Using an automated landmark-based fetal biometry model on pre-selected standard planes, we quantify domain shift and show that training and evaluation confined to a single centre can overestimate performance relative to multicentre testing. To the best of our knowledge, this is the first publicly available multicentre, multi-device, landmark-annotated dataset that covers all primary fetal biometry measures, providing a robust benchmark for studying domain shift and multicentre generalisation and enabling more reliable AI-assisted fetal biometry across centres. All data and annotations are available on the UCL Research Data Repository. Training code and evaluation pipelines are available at https://github.com/surgical-vision/Multicentre-Fetal-Biometry.git.

## Introduction

Fetal biometry is a US-based assessment that measures specific fetal body parts to evaluate growth, determine gestational age (GA), estimate fetal weight, and diagnose prenatal abnormalities. It requires precise measurements obtained by manual identification of anatomical landmarks on standard planes (SPs), predefined cross-sections characterised by orientation, slice position, and presence of specific anatomical structures to ensure standardised fetal assessment across scans and users ^1^. Common SPs include the transventricular (TV) SP to measure fetal head circumference (HC) and biparietal diameter (BPD), the transabdominal (TA) SP to measure fetal abdominal circumference (AC), and the femoral plane to measure femur length (FL). However, manual landmark annotation is time-consuming and highly operator-dependent. In clinical practice, obtaining manual measurements, especially for trainees, results in high inter-operator variability (4.9%–11.1%) and intra-operator variability (3%–6.6%) ^2–4^, contributing to diagnostic uncertainty and hindering reliable fetal growth assessment.

To reduce this human variability and improve workflow efficiency, various approaches for automated fetal biometry have been proposed. These range from single-measurement methods ^5^ targeting individual metrics such as BPD ^6,7^, HC ^8^, and FL ^9^, through SP identification for fetal abdominal views ^10^ that supports downstream biometry, to comprehensive multi-measurement frameworks for fetal weight estimation and landmark-based biometry ^11–15^. While segmentation-based methods ^11,14^ are time-consuming to annotate, direct landmark detection ^16^ better aligns with clinical workflow by requiring sonographers to mark two anatomical points per measurement, representing a faster and more intuitive task ^12^. Recent advances extend beyond task-specific models to end-to-end GA estimation ^17,18^, Bayesian frame aggregation ^19^, real-time temporal validation on US videos ^20^, and large multi-country studies of fetal growth achieving 1.7–2.8 days error across tens of thousands of pregnancies ^21^. In parallel, spectral pooling methods for SP identification ^22^ further improve robustness of plane selection, while foundation models pre-trained on large-scale US corpora ^23,24^ aim to provide reusable representations across tasks and anatomies. Despite this progress, a critical challenge remains: *domain shift*. Various US machines (*e.g.,* General Electric, Philips, Hitachi) and different clinical protocols introduce significant variability. Consequently, models developed and evaluated on limited, single-centre data often perform poorly when deployed across diverse clinical sites. Developing robust models has been hindered by the lack of large-scale datasets capturing this variability across patients, operators, and US devices. While public datasets such as BRATS ^25^ for brain lesion segmentation and FeTA ^26^ for fetal brain segmentation have driven innovation in medical imaging, a recent review of automatic US analysis ^27^ noted that no prior work provides a comprehensive, multicentre, open-access dataset with landmark annotations for all biometric planes (head, abdomen, femur). Table 1 summarises existing datasets for US-based fetal anatomy and biometry, highlighting this gap. Complementary work in intrapartum care, such as the MICCAI 2025 Intrapartum Ultrasound Grand Challenge, provides large multicentre datasets with head–pubic symphysis landmarks for benchmarking end-to-end intrapartum biometry ^28^, but these focus on labour management rather than the antenatal planes targeted in our study.

**Table 1:** Summary of papers describing the fetal US biometry datasets. Note that papers that reuse annotated datasets from previous works are not described here. For all SPs we count both the number of images and fetuses $(\#I/\#F)$.

Paper	Public?	Head SPs	Abdomen SPs	Femur SPs	Crown-rump length SPs	US devices	Methodology
^7^	No	41/41 (training 20/20; test 21/21)	N/A	N/A	N/A	Aplio 780, 790, 500 (Toshiba), Voluson 730 (GE)	Texton-based segmentation with Random Forest classification
^9^	No	273/– (training 106/-; test 167/-)	N/A	321/– (training 124/-; test 197/-)	N/A	Voluson E6, E8, 730, vivid q (GE) Acuson Antares Premium Edition (Siemens) HI VISION Preirus EUB-8500 (Hitachi)	Hybrid Geometric Model-Based Computer Vision with Deformable Model Refinement
^8^	Yes	1334/551 (training 999/806; test 335/335)	N/A	N/A	N/A	Voluson E8, 730 (GE)	Random Forest pixel classification with geometric model-based segmentation
^29^	No	2724/– (training 1948/–; validation 216/–; test 539/–)	N/A	N/A	N/A	Voluson E8 (GE)	DL (FCN-based semantic segmentation with ellipse fitting)
^30^	No	N/A	N/A	436 (augmented to 2,610)/– (training 2300/–; test 310/–)	N/A	Not specified	Random Forest regression (endpoint localization) + DL (SegNet segmentation)
^31^	Partial	1334/551 (HC18) (Training: 999/806; test 335/335)	158/–	315/–	N/A	Voluson E8, 730 (GE) (HC18) Voluson E10 (GE) (local)	DL (Attention MFP-Unet for segmentation) with preprocessing
^12^	No	7274/– (training: 5819/-; validation 1646/–; test: 1455/-)	6717/– (training: 5374/-; validation 2622/- ; test: 1343/-)	7216/– (training: 5773/-; validation 2466/–; test: 1443/-)	3152/– (training: 2522/-; validation 499/–; test: 630/-)	Not specified	DL (caliper removal, classification, segmentation, measurement)
^14^	No	32215/700 (training 19329/420; validation 6443/140; test 6443/140)	26403/700 (training 15842/420; validation 5281/140; test 5280/140)	3706/700 (training 2224/420; validation 741/140; test 741/140)	N/A	Voluson E8, E10, S6, S8, P8 (GE)	Multi-task DL (spatio-temporal video analysis with attention gates and stacked modules)
^11^	No	135/42 (4-fold cross-validation)	103/42 (4-fold cross-validation)	108/42 (4-fold cross-validation)	N/A	Voluson (GE)	DL (multi-class semantic segmentation + ellipse/bbox fitting)
^13^	Yes	999/806 (HC18) 1638/909 (FP)	N/A	761/630 (FP)	N/A	Voluson E8, 730 (GE) (HC18) Voluson E6, S8, S10 (GE), Aloka (FP)	DL (landmark regression with Dynamic Orientation Determination)

To address this bottleneck, this paper presents the first open-access, multicentre, multi-device benchmark dataset of fetal US images with expert anatomical landmark annotations for all clinically used biometric measurements: BPD and occipitofrontal diameter (OFD) acquired on the fetal head plane, transverse abdominal diameter (TAD) and anterior-posterior abdominal diameter (APAD) acquired on the fetal abdominal plane, and FL acquired on the fetal femur plane. The dataset combines three sources: the Fetal Plane (FP) dataset ^1^^,^^1^, the HC18 head dataset ^8^^,^^2^, and an in-house dataset acquired at University College London Hospital (UCLH), which we refer to as UCL; a subset of the UCL data was previously used in AutoFB ^11^. Overall, the dataset comprises 4,513 images from 1,904 subjects, acquired at four clinical sites using seven different US devices. We provide standardised landmark annotations, subject-disjoint train/test splits, and technical validation demonstrating that multicentre training substantially improves generalisation to unseen acquisition settings. We present the technical usability of these annotations for automated fetal biometry within a deep learning (DL) framework ^13^ that is suitable for landmark-based annotation. This study evaluates landmark placement variability exclusively within pre-selected, clinically appropriate SPs, as all images in our dataset were acquired by experienced sonographers who had already identified and captured the correct plane in accordance with ISUOG guidelines ^16^.

## Results

### Quantifying anatomical variability across sites

**Figure 1:**
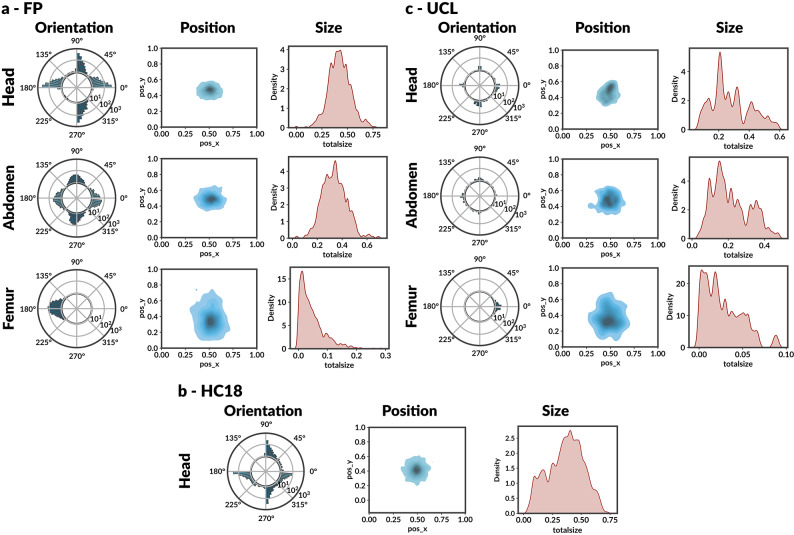
Variability of anatomical structures across (**a**) FP, (**b**) HC18, and (**c**) UCL datasets. Each row represents one anatomical region. *Orientation*: polar histogram with log density scale of measurement angle relative to horizontal axis ([0$^{\circ }$,360$^{\circ }$]); *Position*: 2D kernel density estimation of centre-point location (pos_x, pos_y $\in$ [0,1]); *Size*: 1D kernel density estimation of structure size normalised by image area (unitless). Substantial heterogeneity across datasets reflects realistic clinical variability and demonstrates the domain-shift problem.

We quantified anatomical variability across sites by analysing the structure’s position (centre-point distribution), size (area relative to the image), and orientation (angle from the horizontal). Figure 1 reveals substantial intra- and inter-dataset variability. Notably, structures are inconsistently centred due to differences in operator-dependent framing and probe handling.

Size variability indicates heterogeneous magnification levels, with HC18 exhibiting the tightest range because all images were collected on a single scanner under a controlled protocol. In contrast, FP and UCL include multiple devices and operators. Orientation differences are most evident in the femur plane, where the long bone may appear at any angle depending on fetal pose. These variations reflect the realistic heterogeneity encountered in clinical practice and demonstrate the domain-shift problem: models trained on single-site data fail to generalise to the broader distribution of poses, scales, and orientations in multicentre data. This variability directly accounts for the performance degradation observed in the cross-site evaluation.

### Technical validation on benchmark datasets

To evaluate the usability of the proposed dataset and annotation method, we use BiometryNet ^13^, an end-to-end landmark regression framework for fetal biometry estimation, following the original evaluation protocol. It uses a Dynamic Orientation Determination (DOD) method to enforce measurement-specific orientation consistency during training on a modified HRNet architecture. This provides quantitative evidence of the dataset’s value for developing automated biometry methods.

#### Cross-dataset performance

We performed a comprehensive evaluation across all three datasets and the combined multicentre dataset (MULTICENTRE (M-C)). For each measurement, we quantify localisation error using the Normalised Mean Error (NME) in image space: $\textrm{NME}^{(i)} = (\min \bigl (d_{\textrm{std}}^{(i)}, d_{\textrm{swap}}^{(i)}\bigr ))/(2\,\Vert \textbf{p}_1^{(i)} - \textbf{p}_2^{(i)} \Vert _2)$, where $d_{\textrm{std}}^{(i)}$ and $d_{\textrm{swap}}^{(i)}$ are the sums of endpoint errors under standard and swapped correspondences, respectively, making the metric invariant to endpoint ordering. Table 2 reports the mean and standard deviation of $\textrm{NME}^{(i)}$ across the test set, demonstrating expected domain shift across imaging devices and clinical sites. Although the evaluation metric is endpoint-order invariant, consistent endpoint ordering is still required during training; therefore, we use DOD to harmonise ordering conventions across datasets.

**Table 2:** Cross-data evaluation results showing NME±STD for all train–test combinations across four datasets (FP, HC18, UCL, M-C) and three anatomies. NME is unitless (measurement error normalised by inter-landmark distance). Multicentre models are trained on combined multicentre data. Within each *training dataset block* and for each biometric measurement, bold indicates the best (lowest) NME across the test datasets in that block, and underline indicates the second-best.

Train	Test	NME ± STD				
		Head		Abdomen		Femur
		BPD	OFD	APAD	TAD	FL
FP	FP	**0.03±0.06**	**0.03±0.05**	**0.08±0.06**	**0.08±0.06**	**0.03±0.11**
	HC18	0.08±0.12	0.08±0.13			
	UCL	0.38±0.26	0.22±0.22	0.31±0.23	0.45±0.28	0.90±0.54
	M-C	0.06±0.14	0.05±0.10	0.13±0.15	0.16±0.21	0.12±0.34
HC18	FP	0.06±0.07	0.06±0.07			
	HC18	**0.05±0.09**	**0.04±0.08**			
	UCL	0.15±0.16	0.19±0.23			
	M-C	0.06±0.11	0.07±0.11			
UCL	FP	0.10±0.11	0.09±0.09	0.17±0.13	0.16±0.12	0.07±0.18
	HC18	0.17±0.25	0.13±0.16			
	UCL	**0.08±0.18**	**0.05±0.11**	**0.08±0.14**	**0.08±0.14**	**0.02±0.03**
	M-C	0.12±0.17	0.10±0.12	0.15±0.14	0.14±0.13	0.06±0.17
M-C	FP	0.03±0.05	**0.03±0.04**	0.08±0.06	0.09±0.07	0.03±0.10
	HC18	0.05±0.08	0.04±0.07			
	UCL	**0.02±0.02**	0.03±0.11	**0.05±0.12**	**0.05±0.12**	**0.01±0.01**
	M-C	0.04±0.07	0.03±0.06	0.07±0.08	0.08±0.08	0.03±0.09

**Figure 2:**
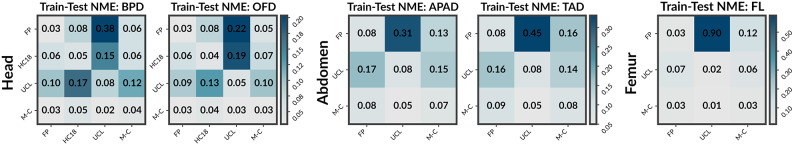
Cross-dataset generalisation heatmaps for fetal biometry. Train$\rightarrow$Test NME (lower is better) for each biometric measurement. Rows denote the training dataset and columns the test dataset. For the abdomen and femur, HC18 is omitted where results are unavailable in the cross-dataset evaluation table. Cell values report mean NME. Colour scales are shared within each anatomy group (Head: BPD/OFD; abdomen: APAD/TAD; Femur: FL).

Figure 2 shows train–test NME heatmaps, with rows indicating the training dataset and columns the test dataset. This representation highlights the relative robustness of M-C training across test datasets and the pronounced asymmetry of some cross-domain pairs, particularly for FL.

Within-domain performance (training and testing on the same dataset) achieved low errors across all anatomies, with NME values typically below 0.1 for head and abdomen (BPD, OFD, APAD, TAD) and below 0.05 for FL (Table 2). Cross-domain performance (training on one dataset, testing on another) showed a consistent increase in NME, particularly when source and target differed in device manufacturer or acquisition protocol. Averaged across all train–test pairs, head and abdomen NME roughly doubled relative to within-domain performance, while femur NME reached as high as 0.90 in the FP$\rightarrow$UCL setting, indicating substantial domain shift.

For head biometry (BPD and OFD), the M-C model achieved the best overall cross-site generalisation. When trained on M-C and tested on UCL, it achieved NME = 0.02±0.02 for BPD and 0.03±0.11 for OFD, outperforming even the UCL-trained model evaluated on its own test set (0.08±0.18 and 0.05±0.11, respectively). After the preprocessing to ensure that all landmarks fall within the heatmap, HC18-trained models exhibit competitive within-domain performance and moderate cross-domain degradation. For abdomen biometry (APAD and TAD), UCL- and M-C-trained models achieved the lowest within-domain errors (UCL$\rightarrow$UCL: 0.08±0.14 for both APAD and TAD; M-C$\rightarrow$M-C: 0.07±0.08 and 0.08±0.08). Cross-domain transfer between FP and UCL showed a clear but more modest degradation than for the femur, with NME increasing to 0.16–0.31 depending on the direction of transfer. For femur length, both FP and UCL models achieved excellent within-domain performance (NME $\approx$ 0.03), but cross-domain transfer remained challenging, especially from FP to UCL (FP$\rightarrow$UCL: 0.90±0.54). The M-C-trained model achieved the lowest femur errors on the M-C test set (0.03±0.09), but cross-site generalisation for FL remains limited.

**Figure 3:**
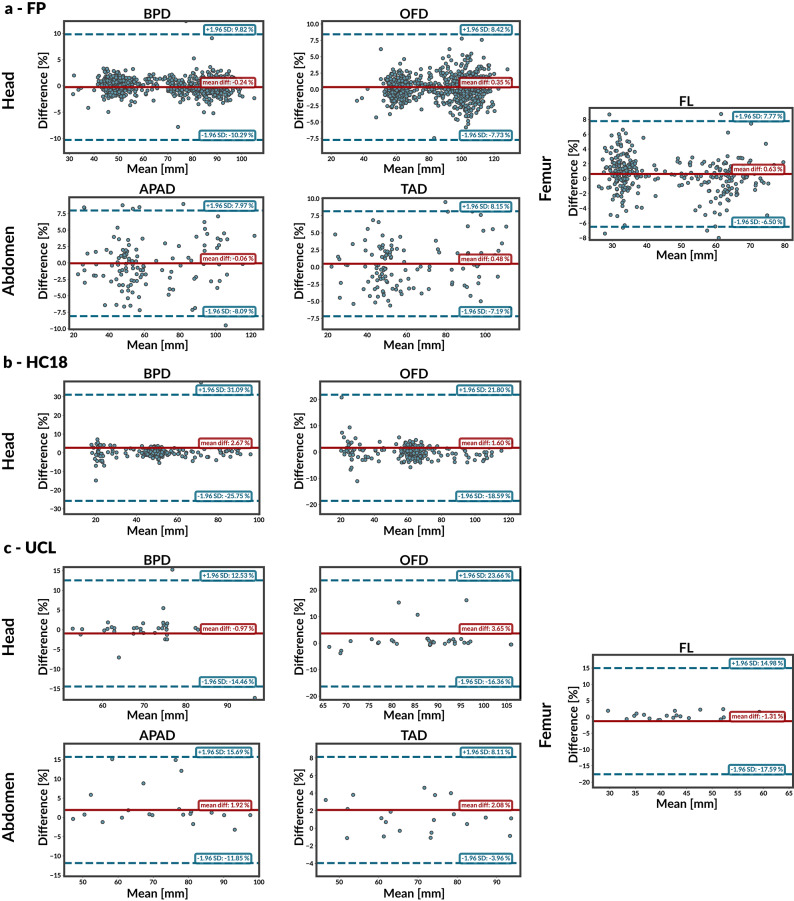
Normalised Bland–Altman agreement plots between BiometryNet predictions and ground-truth fetal biometry measurements for (**a**) FP, (**b**) HC18, and (**c**) UCL datasets. Horizontal axis: mean measurement (mm). Vertical axis: percent difference relative to the ground-truth measurement. Solid lines indicate the mean bias and dashed lines the 95% limits of agreement (bias $\pm 1.96$ SD).

Figure 3 shows the normalised Bland–Altman plots for each dataset, illustrating the agreement between predicted and ground-truth measurements as a percent difference relative to the ground-truth value. Expressing errors as percentages mitigates heteroscedasticity with increasing measurement size and enables comparison across anatomies. Solid lines indicate the mean bias and dashed lines indicate the 95% limits of agreement (bias ± 1.96 SD). For each landmark pair, ground-truth and predicted distances are converted from pixels to millimetres; in the Bland–Altman analysis, the x-axis shows the mean measurement in millimetres and the y-axis shows the percentage difference relative to the ground truth.

**Figure 4:**
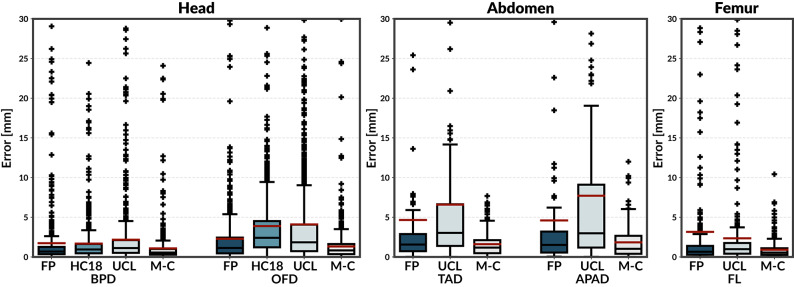
Absolute biometry error (mm) on the M-C test set for models trained on FP, HC18, UCL, and M-C datasets. Boxplots are shown for Head (BPD, OFD), Abdomen (TAD, APAD), and Femur (FL). The y-axis is truncated at 30 mm to improve visualisation of the central distribution.

Figure 4 shows the absolute biometry error (mm) on the M-C test set for models trained on FP, HC18, UCL, and M-C datasets for Head (BPD, OFD), Abdomen (TAD, APAD), and Femur (FL). To focus on the central error distribution, the y-axis is truncated at 30 mm; a small number of larger outliers fall above this range. The M-C-trained model achieves competitive medians across all anatomies with consistently narrow interquartile ranges, indicating robust and stable performance. In contrast, HC18-trained models exhibit wider error distributions on the M-C test set, reflecting residual domain shift despite the corrected preprocessing. For the head, the median errors of the M-C model (approximately 0.2 mm for BPD and OFD) correspond to clinically acceptable GA variation in late pregnancy.

Overall, these results demonstrate that models trained on multicentre data generalise more robustly across clinical sites, establishing baseline performance benchmarks for future method development and highlighting the importance of multicentre training for clinically deployable fetal biometry systems.

### Data description

**Figure 5:**
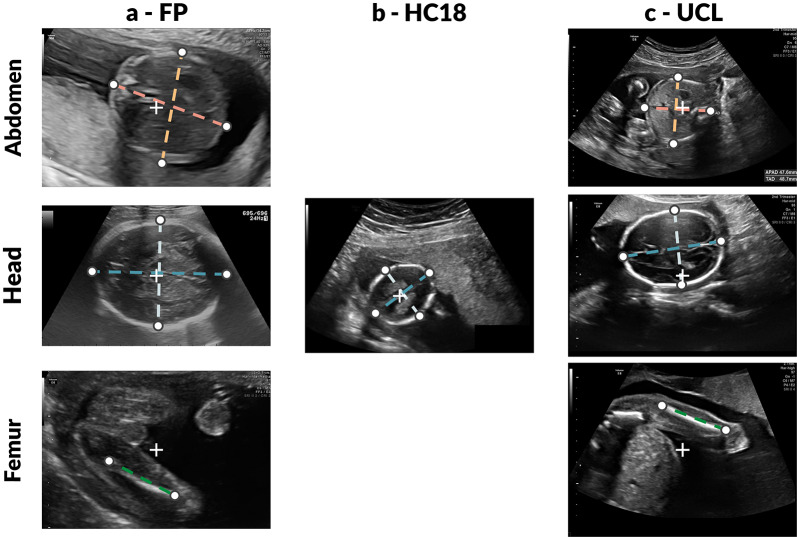
Example raw fetal US SPs from the (**a**) FP, (**b**) HC18, and (**c**) UCL datasets before preprocessing. Each row corresponds to an anatomical plane (head, abdomen, femur); HC18 contains head images only. Overlaid landmark endpoints and connecting lines indicate the annotated biometric measurements, highlighting differences between manual calliper-style annotations used in FP and UCL, and ellipse-derived annotations in HC18, as well as cross-dataset variation in image appearance, field of view, and acquisition characteristics.

The dataset comprises three subsets (Table 3) totalling 4,513 images from 1,904 subjects across four clinical sites and seven ultrasound devices. HC18 spans all trimesters, with a clinical screening bias toward the second trimester. In contrast, FP and UCL datasets are predominantly in the second/third trimester (14–40 weeks), encompassing the GA range used for fetal weight estimation. This diversity ensures the benchmark covers the full clinical spectrum of fetal biometry assessment. Figure 5 shows representative SPs from each dataset, illustrating the visual variability in image appearance, field of view, and annotation style across sources. When combined for multicentre training, we refer to the unified dataset as M-C.

#### File organisation

The repository is organised into two top-level directories, images/ and annotations/, each containing per-dataset subdirectories. Dataset-specific README files at the root level document acquisition protocols, device details, imaging parameters, annotation conventions, and quality control procedures; a general README provides an overview and guidance on selecting datasets. The repository follows this structure:
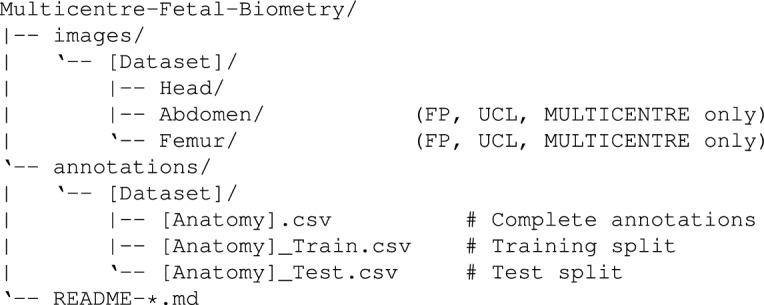


where [Dataset] and * are one of {FP, HC18, UCL, M-C}, [Anatomy] is one of {Head, Abdomen, Femur}.

#### Annotation format

Annotations are provided as CSV files, each containing landmark coordinates for a single measurement. All CSV files include common fields *index* (sequential index in the dataset), *image_name* (filename of the corresponding US image), *scale* (scaling factor applied during preprocessing), *centre_w, centre_h* (centre coordinates of the region of interest), *px_to_mm_rate* (pixel-to-millimetre conversion rate), *mm_dist* (distance marker value visible in the image, typically 5mm or 10mm), *Algo* (device or algorithm identifier), *SubjectID* (de-identified subject identifier), *Split* (data split indicator, train or test).

**Head measurements (BPD and OFD)**: bpd_1_x, bpd_1_y, bpd_2_x, bpd_2_y (landmarks for biparietal diameter) and ofd_1_x, ofd_1_y, ofd_2_x, ofd_2_y (landmarks for occipitofrontal diameter).

**Abdomen measurements (TAD and APAD)**:tad_1_x, tad_1_y, tad_2_x, tad_2_y (landmarks for transverse abdominal diameter) and apad_1_x, apad_1_y, apad_2_x, apad_2_y (landmarks for anterior-posterior abdominal diameter).

**Femur measurements (FL)**: fl_1_x, fl_1_y, fl_2_x, fl_2_y (landmarks for femur length, calculated between proximal and distal ends).

#### Data splits

All subsets provide standardised, subject-disjoint train/test splits using a fixed hold-out strategy to ensure unbiased and reproducible evaluation. No cross-validation is employed; each dataset has a single, predetermined split. Images from the same subject appear in only one split (either training or test set), preventing data leakage. For FP and HC18, the splits are inherited from BiometryNet ^13^, which established a landmark-based benchmark on these datasets. For UCL, the split is derived from the 4-fold cross-validation structure used in AutoFB ^11^: folds 1–3 form the training set and fold 4 serves as the test set, preserving subject-disjoint partitioning. Images in the expanded UCL dataset (424 images from 51 pregnancies, compared with the original 346 images from 42 pregnancies in AutoFB) that were not present in the original folds were distributed evenly between training and test sets while maintaining subject disjointness. The split assignments are provided in separate files ([Anatomy]_Train.csv and [Anatomy]_Test.csv) and indicated in the Split column of the complete annotation files ([Anatomy].csv).

## Discussion

We present the first comprehensive, open-access, multi-device, multicentre dataset for automated landmark-based fetal biometry, together with open-source training and evaluation code. By combining 3 sources (FP, HC18, UCL) across 7 US devices and 4 clinical sites, we provide 4,513 annotated images that capture realistic clinical variability. Unlike single-anatomy (HC18) or single-site (AutoFB) datasets, ours enables systematic quantification of domain shift, a critical barrier to clinical deployment of artificial intelligence (AI)-based biometry systems. Our dataset and experiments focus on landmark detection within pre-validated SPs, and the variability introduced by SP selection was not quantified in this work. While this approach isolates variability in landmark placement for rigorous benchmarking, a fully automated pipeline would need to address either plane verification or robustness variations in planes. Future work should extend benchmarking to include raw US sweeps, near-boundary planes, and non-standard views to quantify the variability introduced by SP selection, which remains one of the largest sources of measurement error in clinical practice and is not captured by our current benchmark. Incorporating raw video sequences would additionally enable evaluation of end-to-end pipelines that jointly perform plane selection and biometry ^32^.

For the FP and UCL datasets, the pixel-to-millimetre scale is recovered from the on-screen ruler rather than directly from machine-embedded calibration data. Independent validation of the recovered scale against DICOM-embedded calibration values, where available, would further confirm its accuracy. However, because the same pixel-to-millimetre conversion factor is applied to both ground-truth and predicted measurements, any residual systematic bias is expected to affect them equally and is therefore unlikely to alter the relative error metrics and comparative conclusions reported in this work.

Table 2 quantifies the performance gap between within-dataset and cross-dataset evaluation. Within-domain evaluation achieved low NME values (typically below 0.1 for head and abdomen measurements), whereas cross-domain evaluation showed a marked increase in error, with some train–test pairs more than doubling the NME and femur cross-domain NME reaching values close to 1.0 in the most challenging settings (e.g., FP$\rightarrow$UCL). This domain shift arises from differences in imaging devices (GE Voluson E8 vs. Aloka), operator framing conventions (Figure 1), and variability in fetal presentation.

Measurement accuracy varies with the GA. In the HC18 study ^8^ the error in GA estimation from HC measurements increased from the first trimester (0.6±4.3 days) to the third (2.5±12.4 days), corresponding to ${\sim }$0.7% and ${\sim }$1.1% of the mean GA at each trimester, respectively, reflecting increased biological variability and reduced sensitivity of HC to GA at later stages. FP and UCL datasets capture this real-world GA variability across multiple devices and operators, though trimester-specific error stratification was not quantified in the original studies.

The M-C model achieved the lowest mean NME when tested on UCL data (BPD: 0.02±0.02; OFD: 0.03±0.11), outperforming the UCL-trained model tested on its own data (BPD: 0.08±0.18; OFD: 0.05±0.11). This suggests that incorporating diverse training data can improve generalisation even to datasets included in the training set; however, with only 49 UCL test images, larger multicentre test sets would be needed to confirm this trend. The M-C model showed limited cross-site generalisation for femur measurements on the M-C test set (0.03±0.09), and inspection of the predictions revealed that many high-NME failures correspond to the model selecting the wrong femoral instance when more than one long bone is visible in the image, rather than misplacing endpoints along the correct bone. Since NME is normalised by the ground-truth inter-landmark distance (approximately the femur length), an error on the order of the distance between the two femurs produces NME values close to 1.0. Thus, the heavy tail in cross-domain FL errors reflects instance ambiguity rather than small-scale localisation noise, indicating that robust femur biometry may require anatomy-specific strategies. Potential approaches include computing network uncertainty from output heatmaps to reject ambiguous instances, using region-of-interest priors to constrain the search space to a single femoral instance, multiple-instance learning to explicitly model the presence of multiple candidate bones, or auxiliary classification heads that distinguish left from right femur before landmark regression.

Harmonising data across sites also required overcoming inconsistent annotation protocols. For instance, differing endpoint ordering conventions across datasets initially inflated cross-domain errors, underscoring the need for orientation-invariant evaluation and training mechanisms, such as the DOD module we employed. Besides, the HC18 dataset showed markedly inferior cross-domain generalisation due to landmark-space mismatch before preprocessing. After applying image-centric preprocessing parameters, HC18-trained models show competitive within-domain performance and moderate cross-domain degradation (Table 2). Although this annotation protocol difference, including inner-outer versus outer-outer conventions, could introduce systematic bias in cross-domain comparisons involving HC18, indirect evidence suggests that domain shift is the dominant error source. For femur measurements, FP and UCL share the same manual protocol, and HC18 contributes no femur data; still, cross-domain FL errors remain the largest across all measurements, ruling out annotation differences as a confound. Furthermore, as shown in Figure 2 for head measurements, models trained on FP generalise better to HC18 (cross-protocol) than to UCL (same protocol). If annotation protocol were the dominant source of error, we would expect cross-protocol transfers (FP$\leftrightarrow$HC18) to perform consistently worse than same-protocol cross-domain transfers (FP$\leftrightarrow$UCL); the data show the opposite pattern, indicating that domain shift, rather than annotation protocol differences, is the primary driver of cross-domain degradation. A controlled ablation with dual-protocol annotations on a shared image subset would be needed to fully disentangle these effects, and is recommended as future work.

These findings have important implications for clinical deployment. The marked performance degradation from within-domain to cross-domain settings indicates that models trained on single-site data are unlikely to maintain accuracy when deployed in different clinical settings with varying US devices and operator practices. The improved cross-site generalisation of multicentre models (e.g., M-C$\rightarrow$UCL, achieving significantly lower NME values than FP$\rightarrow$UCL for head biometry) suggests that robust clinical deployment will require training on diverse, multi-source datasets that capture real-world variability. For sonographers, successful automation could reduce measurement time and inter-operator variability (currently 4.9–11.1% across observers) ^4^, but only if models are validated across the full spectrum of clinical acquisition conditions encountered in practice.

By releasing this dataset publicly, we enable the research community to develop and benchmark automated fetal biometry methods under realistic clinical variability, addressing reproducibility challenges that have limited progress in this field  ^33^. Our benchmarking infrastructure with standardised splits and training and evaluation code provides a foundation for reproducible research. Future work should extend this infrastructure in three directions: i) temporal sequences and video data to leverage anatomical motion consistency, ii) pathological cases and anomalous presentations to ensure robustness across clinical scenarios, and iii) additional anatomical planes (e.g., fetal thorax, spine, or maternal cervical length) to enable comprehensive fetal assessment. By enabling robust, generalisable benchmarking across diverse clinical settings, this work lays the foundation for future advances in automated fetal biometry and improved clinical screening workflows.

## Methods

Our approach combines three existing datasets with different acquisition protocols, devices, and annotation strategies. We standardise annotations to a landmark-based format with consistent ordering conventions, compute image-specific preprocessing parameters for the model input, and provide subject-disjoint training and testing splits for reproducible benchmarking. This section describes each source dataset, our annotation pipeline, preprocessing methodology, and the scale-recovery method for converting pixel measurements to clinical units.

### Source datasets

We use two publicly available datasets, FP ^1^ and HC18 ^8^, and UCL, an in-house dataset from UCLH (Table 3).

**Table 3:** Summary of the datasets included in this study. Each dataset contains standard 2D US planes of the fetal head, abdomen, and femur, annotated with anatomical landmarks for biometry estimation, except for HC18, which only contains the fetal head.

Dataset	Source/institution	#Subjects	#Images	Anatomical planes	Split (Train/test)	Notes
**FP** ^1^	Vall d’Hebron and Hospital Sant Joan de Déu, Barcelona	1,047	3,090	Head, Abdomen, Femur	Head: 757/880; Abdomen: 568/125; Femur: 437/323	Landmark annotations by obstetricians.
**HC18** ^8^	Radboud University Medical Center, Netherlands	806	999	Head only	Head: 737/262	BPD and OFD derived via least-square ellipse fitting from head circumference masks.
**UCL**	University College London Hospital, UK	51	424	Head, Abdomen, Femur	Head: 110/49; Abdomen: 94/36; Femur: 96/39	Approved under IRAS ID 230125. Pseudo-anonymised; NHS Fetal Anomaly Screening Programme compliant. Landmark annotations by obstetricians.
**M-C**	Multicentre (all the above)	**1904**	**4513**	**Head, Abdomen, Femur**	**Head: 1604/1191; Abdomen: 662/161; Femur: 533/362**	

FP ^1^ was originally designed for the US SPs classification challenge. The US images were acquired on six US devices: three GE Voluson E6, one Voluson S8, one Voluson S10, and one Aloka at two clinical sites in Barcelona, Spain. Since not all images in FP qualified as SPs for fetal biometry ^16^, we selected 1637 (909 subjects) fetal head, 693 (586 subjects) fetal abdomen, and 760 (629 subjects) fetal femur SPs. Many subjects contributed images across multiple anatomies, resulting in a total of 3090 images from 1047 unique subjects. An obstetrician then manually annotated the landmarks on each image with the VGG Image Annotator (VIA) annotation tool ^34^. HC18 ^8^ was designed for the fetal HC challenge. The US images were acquired with two US devices, GE Voluson E8 and 730, at a single clinical site in the Netherlands. All HC18 data, 999 (806 subjects) fetal head SPs, were annotated with the HC measurement. We extracted the BPD and OFD biometric measurements from an ellipse’s major and minor axes by least-square fitting ^35^ onto the ground-truth mask. For the UCL dataset, the local research ethics committee reviewed and approved the collection process (IRAS ID 230125). Patients attending the hospital for routine US examination were enrolled and pseudo-anonymised by the clinical research staff. The hospital protocol followed the NHS Fetal Anomaly Screening Programme^3^. The inclusion criteria were: i) pregnant patients attending UCLH for routine second- or third-trimester US screening (14–40 weeks GA); ii) written informed consent; and iii) US images acquired on standard anatomical planes (head, abdomen, femur) following ISUOG guidelines ^16^. No exclusions were applied based on fetal pathology, plurality, or maternal characteristics; the dataset reflects the clinical population attending UCLH during the study period. Images that did not correspond to a recognisable SP for fetal biometry, with severe artefacts or occlusions that prevented reliable landmark identification, or with absent or unreadable on-screen scale rulers, were excluded during the selection process. The complete image library from each US examination session was transferred to the research database. All US images saved by the operator were considered optimal for that scan and of diagnostic quality. The operator applied the measurement callipers, and in most cases, images with and without the callipers were saved. A total of 424 images were included from 51 pregnancies, expanding the original 346-image cohort from 42 pregnancies used in AutoFB ^11^. Each image in the dataset was classified as AC, HC, or FL. The VIA annotation tool ^34^ was used to annotate the head, abdomen, or femur within each image. The fully anonymised US SP images obtained exhibit significant intra-class variability. For example, in some cases the femur is well aligned with the plane’s centre, while in others it appears as a small, distant object.

### Data preprocessing and standardisation

All images underwent standardised preprocessing to ensure consistency and reproducibility across different US devices and acquisition protocols.

#### Annotation protocols

Two complementary annotation approaches were used across datasets. This methodological difference (manual vs. ellipse-derived) contributes to the observed performance variations and underscores the importance of harmonising the annotation protocol in multicentre datasets.

**Manual landmark annotation (FP and UCL)** Expert sonographers manually placed anatomical landmarks using the VIA tool ^34^, following ISUOG guidelines ^16^. FP annotations were performed by senior maternal-fetal clinicians (inter-rater agreement 93–95% ^1^); UCL annotations were performed by a clinical research fellow under the supervision of a senior consultant. Each measurement requires two points marking the start and end of the biometric diameter; on average, each plane annotation for landmarks took 20 seconds, which is lower than the 70 seconds required for manual structure segmentation delineation ^11^.

**Ellipse-derived landmarks (HC18)** Landmarks were automatically extracted from expert-annotated HC segmentation masks via least-squares ellipse fitting ^35^. BPD and OFD measurements were derived from the ellipse’s minor and major axes, respectively. Inter-observer variability in the HC18 test set (experienced sonographer vs. medical researcher) showed GA estimation differences of 0.8–1.6 days, highlighting the inherent variability even between trained observers ^8^. This ellipse-fitting approach differs from clinical calliper placement conventions, introducing potential systematic bias in HC18 annotations.

All landmark coordinates are provided in pixel coordinates, with the origin (0, 0) at the top-left corner of the image. Coordinates are provided as floating-point values to maintain subpixel precision after scaling operations. Quality control measures ensured annotation accuracy: i) plane verification: each image verified to show the correct SP for the intended measurement; ii) anatomical checks: presence of required landmarks confirmed (e.g., cavum septum pellucidum for head, stomach bubble for abdomen); iii) measurement validation: computed measurements checked against expected ranges for gestational age; iv) outlier detection: automated checks identified potential annotation errors for manual review.

#### Preprocessing and training setup

For technical validation and to provide a strong baseline, we adopt BiometryNet ^13^ as our landmark regression model. BiometryNet is based on an HRNet-W18 backbone with DOD and achieved state-of-the-art performance on the HC18 challenge, making it a natural reference for single-site landmark-based fetal biometry. Using the publicly available implementation, we retain the core architecture and training strategy, while adapting preprocessing, augmentation, and cross-dataset training to our multicentre setting as described below.

All US images underwent standard preprocessing before model training and evaluation to ensure consistency across datasets and compatibility with downstream DL frameworks. Each image was first converted to grayscale and cropped to remove any textual overlays, scale bars, or machine-interface elements, following the same strategy used in AutoFB ^11^. Pixel intensities were normalised to the range [0, 1] using linear min–max scaling. For model input, the HRNet-W18 landmark detection model requires fixed-size 256 $\times$ 256 pixel inputs. For each image, we compute preprocessing parameters as follows: the crop centre is set to the image centre (*w*/2, *h*/2), and the scale factor is initially calculated as $\texttt {scale} = \max (w, h)/(1.7 \times 256)$, where *w* and *h* are the image dimensions. This ensures the entire image content is visible within the crop while maintaining consistent preprocessing across datasets. The HC18 dataset was originally preprocessed using ellipse-centric parameters centred at the ellipse centroid, with scale derived from the ellipse dimensions, resulting in 84.5% of landmarks falling outside the 64$\times$64 heatmap space used to generate targets. We therefore recomputed the HC18 crop parameters by recentring at the image midpoint and setting the scale from the maximum landmark-to-centre distance with an additional safety margin, which ensured that all annotated landmarks lay well within the valid 64$\times$64 heatmap area. The same verification procedure was then applied uniformly across all datasets. For each image, we checked that all landmark coordinates, after affine transformation into the $64\times 64$ heatmap space used for target generation, fell within valid bounds. If any landmarks still fell outside the heatmap after the default computation described above, the scale was progressively increased until all landmarks were mapped to valid heatmap indices within a small safety margin from the borders. This automated, deterministic correction affected only a small fraction of images and ensured that all ground-truth target heatmaps contained valid Gaussian peaks, eliminating silent training failures caused by out-of-bounds landmarks. Cross-dataset training revealed that different datasets employed opposite endpoint conventions for specific measurements. To ensure consistent supervision, we leveraged BiometryNet’s DOD mechanism, which learns a measurement-specific orientation vector $\mathbf {d\_vect}$ during training and extended the framework to save $\mathbf {d\_vect}$ and apply it at inference time, which automatically corrects predicted landmarks to match the training dataset’s endpoint ordering convention. This approach avoids manual point-swapping and instead allows the model to learn and remember the appropriate orientation for each measurement.

For data augmentation, random transformations were applied during training to increase robustness to operator- and device-related variations. These included random rotations within $\pm 20^{\circ }$, horizontal and vertical flips, random scaling (90–110%), translation (up to 10% of the image size), and brightness/contrast jitter (up to 20%). All augmentation parameters and intensity normalisation follow the settings reported in AutoFB ^11^, which demonstrated effective regularisation for heterogeneous US data.

Each model was trained for 200 epochs with a batch size of 16 using the Adam optimiser with an initial learning rate of $1\times 10^{-4}$ on a single Tesla® NVIDIA A100-SXM4-40GB GPU. The learning rate was reduced by a factor of 0.2 at epochs 10, 40, 90, and 150 using a multi-step schedule. No early stopping was applied; training ran for the full 200 epochs to ensure convergence across all dataset configurations. All models were initialised from ImageNet-pretrained HRNet-W18 weights.

### Scale recovery methodology

Conversion from pixels to millimetres (px_to_mm_rate field in the annotation files) is required to obtain accurate measurements that can be compared with those obtained in the clinic. While this information is usually available during an examination or is embedded in the raw image data, some retrospectively collected images may lack it. We perform scale recovery using the approach presented in AutoFB ^11^, which exploits the consistent visual interface of the US machines. For GE Voluson devices (used in all three source datasets), the ruler visible on the left-hand side of the US image is detected using normalised cross-correlation template matching. Templates corresponding to the small (5 mm) and large (10 mm) ruler tick marks are matched against the left-margin band of the image. The pixel spacing between consecutive detected markers is computed and averaged to obtain the conversion factor. For Aloka devices, where not all ruler markers are displayed, we use an alternative approach: markers are detected on the lower vertical band by analysing intensity gradients, and the scale is estimated from the median inter-tick pixel spacing, assuming a 5 mm tick interval, which was confirmed by manual visual inspection of the scale against expected fetal anatomical sizes. For the FP and UCL datasets, scale recovery was successfully applied to all images, whereas for the HC18 dataset, the pixel-to-millimetre scale was provided directly by the dataset creators, so scale recovery was not required. The method is robust across the seven device types in our dataset because all medical-grade US machines display standardised ruler markers with consistent visual appearance. We note that the pixel-to-millimetre conversion factor is used identically for both ground-truth and predicted measurements.

## Data Availability

The fetal ultrasound images and landmark annotations generated in this study are available on the UCL Research Data Repository at https://doi.org/10.5522/04/30819911. Training code and evaluation pipelines are available at https://github.com/surgical-vision/Multicentre-Fetal-Biometry.

## Footnotes

1 FETAL PLANES: Common maternal-fetal ultrasound images (Accessed December 2025)

2 HC18 - Automated measurement of fetal head circumference using 2D ultrasound images (Accessed December 2025)

3 NHS Fetal Anomaly Screening Handbook (Accessed March 2026)
